# Sixth Annual Session of the Tri-State Medical Society of Alabama, Georgia, and Tennessee, at Atlanta

**Published:** 1894-11

**Authors:** 


### *SOCIETY REPORTS.*

### SIXTH ANNUAL SESSION OF THE TRI-STATE MEDICAL SOCIETY OF ALABAMA, GEORGIA, AND TENNESSEE, AT ATLANTA.

### FIRST DAY, TUESDAY, OCTOBER 9, 1894.

Opened with prayer by REV. HENRY McDONALD.

Address of welcome was made by WILLIS F. WESTMORELAND, Atlanta, and responded to by R. M. CUNNINGHAM, Birmingham, Ala.

DR. J. C. LEGRAND read a paper on "The Responsibility of a Class of Criminals from a Medico-Legal Point of View," in which he advocated castration for capital crimes instead of hanging. The object of this is two-fold: First, as a punishment and reformatory measure; second, to prevent the increase of the criminal class. He did not believe that hanging has the deterring effect that castration would have. This treatment cannot be applied except to those convicted of capital crime.

DR. R. M. CUNNINGHAM said: External influences alone do not make or prevent criminals. Under precisely the same environment of law—social, ethical, religious, and moral,—we have some committing crime and others not. Why so? It is due to some internal, personal agency, that stimulates to the commission of crime, or that restrains the moral inhibition of crime—in either case interrupting the mental, moral, and physical equipoise which in one insures honesty and obedience to law and order, and in the other the violation of same. Whence comes the principle? Partly from race, partly from nationality, and very largely from family. I think the real cause of crime is found in the criminal himself. When such a principle is discovered, it is the duty of the law to protect society against the propagation of this principle, by emasculation of the criminal, and thereby prevent any posterity.

DR. J. B. COWAN said: There is no diversity of laws of civilized countries in regard to rape. It is always ranked with murder. The tendency to crime is not alone in the man, but also due to environment. The question takes us into the domain of theology. The rapist is insane. He is irresponsible.

DR. L. P. BROUILLETTE read a paper on "Treatment of Stricture of the Urethra by Electrolysis," in which he reported a case in which the urethra was impermeable. There was no pain or interference with business. The treatment is limited to stricture due to inflammatory action. He quoted Neuman, as to method, differing in that he gave séances on alternate days. He insists on the permanency of the cure when properly administered.

DR. J. B. COWAN had had no bad results from the older methods. All cases had been relieved and most cured. He would be unwilling to throw away the knife in all cases.

DR. F. W. MCRAE was opposed to the method, mainly through the report of Dr. Keyes of New York, who states that the results are no better than by rapid dilatation. Had no personal experience with the method. His method was to incise the stricture, and after healing to dilate. The New York Academy of Medicine appointed a committee to investigate the question, and after a year they reported the method a failure.

A paper was read by FRANK TRESTER SMITH, "The Induction of Labor to Prevent Blindness." The paper was mainly historical and advocated the induction of labor when blindness was threatened, because this indicated that there was kidney disease of so grave a character as to threaten life of both mother and fetus.

DR. J. A. GOGGANS said that he had seen a number of cases with albuminuric retinitis and they usually went blind and died.

DR. J. B. COWAN said that we could not be sure that the albuminuric retinitis was due to the pregnant state. He did not believe in inducing labor promiscuously.

DR. SMITH, in closing the discussion, said, that when we had a case of increasing blindness in the pregnant state, we could be quite sure that it was due to the pregnancy. This was more certain if the blindness was increasing rapidly, and especially if in previous pregnancies there had been attacks of blindness.

A paper by E. VAN GOIDTSNOVEN was read on "Slaughter of the

Innocents," taking the ground that the child ought never to be deliberately killed, condemning the induction of labor and craniotomy, and advocating Cæsarean section and Porro's operation.

DR. R. R. KIME said that we should be careful from a moral standpoint. He did not condemn *in toto* the induction of abortion for the vomiting of pregnancy; still it was getting too common. Instead of a craniotomy, a Cæsarean operation, a symphyseotomy, or a Porro's operation. In some cases of kidney affections with uremic symptoms, if we wait, the mother will be dead as well as the child.

DR. R. M. CUNNINGHAM could not agree that there were no cases where he should induce artificial labor. The relation between fetus and mother was different from that between child and mother. The fetus was simply a vitalized neoplasm, and should be removed only to preserve life. Craniotomy is an operation that should be obsolete and is with all scientific men.

DR. W. E. B. DAVIS said that those who had performed the most successful operations advocated that in extra-uterine pregnancies early operations should be performed. They urge this to save the mother's life.

DR. FRANK S. PARSONS read a paper on "Pneumonia in Children." The following is a synopsis:

Pneumonia in children considered under two great classes, croupous and catarrhal. The first considered as specific, infectious, and self-limited, which should be classed as zymotic. The self-limitation appears due to an antipneumotoxin, which may some day be used as a therapeutic agent in curing or aborting this variety of pneumonia, but nothing at present seems efficient in aborting true pneumonia. Treatment in children should be based on the following lines:

1. Promotion of diaphoresis, and elimination of carbonic acid gas.

2. Promotion of elimination through the kidneys.

3. Promotion of alkalinity of blood-lessening tendency to fibrin formation.

4. Promotion ot rest.

*Treatment of Temperature, Cardiac Debility, and Nervous System.*—Expectorants, purgatives and external applications are considered briefly.

*Catarrhal, or Broncho-Pneumonia.*—Entirely different from croupous variety, and should be considered a separate disease. Hygiene of these cases, chloral hydrate, as an antipyretic. Treatment of the cough, an expectoration. Stimulation. Causes of broncho-pneumonia should occasionally be sought in the intestinal canal.

DR. R. R. KIME read a paper entitled "Essentials of Obstetric Nursing." Asepsis on the part of physician, patient, and nurse is required. Asepsis may be attained by cleanliness or antiseptics. It is essential that the physician and nurse have clean clothing, hands, and finger nails, and everything coming in contact with patient be rendered aseptic and kept so during the puerperium, even the patient herself. Advised washing and boiling of all cloths, towels, and bedding to be used about the patient. Advised use of antiseptic vulval pad with abdominal binder. Condemned vaginal douche after normal labor Advised use of catheter by sight after cleansing parts, and not by touch, so as to prevent infecting bladder. Condemned astringents to nipples before labor. Believed infection is more common than most physicians admit, in both city and country practice. That the majority, not all, cases of infection can be prevented by due diligence on the part of the physician and nurse. That many cases of mild infection pass unnoticed by the physician, because they are not attended with severe constitutional disturbances, or are classed as malaria, milk fever, taking cold, inflammation of bowels, typhoid fever, etc.

DR. F. S. PARSONS agreed in the main with the author, except that he thought that so much antiseptic material was not necessary. Hot water was the best antiseptic, and all that was needed ordinarily. He used less antiseptics afterwards, but placed the patient in a semi-sitting posture. He advocated immersing the child in warm water and washing it under water. To prevent excoriation of the naval he wrapped the cord in absorbent cotton. Over this he placed some adhesive plaster. Did not use a binder in any form.

DR. J. A. GOGGANS read a paper on "The Surgical Treatment of

Empyema." The methods are: aspiration, rib resection, and the open method. The natural tendency is more favorable in children. Empyema is a symptom of various diseases—not a disease itself. Aspiration has many advantages in childhood. He reported thirteen cases operated on, mostly by aspiration. He advocated the operation of Porro. The conclusions were as follows:

1. That it is useless to delay the operation of thorocotomy, especially after finding that the pus contains the bacillus of tubercle, or is the result of microbes being introduced through the lymphatics, or when the empyema is developed in cases of general pyemia.

2. That the opening should be made above the ninth rib.

3. That antiseptic injections should be used only in the above mentioned varieties of empyema.

DR. J. M. MATTHEWS read a paper on "Some Points in Rectal Surgery." Fistula in ano is one of the most important of diseases of the rectum. It requires more delicate and precise surgery than most surgical affections. Irritable ulcer or fissure can be cured by divulsion of the sphincter. At the International Medical Congress, at Washington, he read a paper on "The Anatomy of the Rectum in Relation to Reflexes," in which he endeavored to show that much elucidation could be thrown upon many suspected diseases by tracing their origin to disease of the rectum. This idea has been run away with by the so-called "orificial" surgeons, who have done much harm by their method of removing an inch or two of the lower part of the bowel. This practice cannot be too severely condemned.

In ulceration of the rectum, sixty per cent. are due to syphilis. Simple ulceration is extremely rare. In cancer of the rectum, lumbar colotomy is to be preferred to inguinal. When practicable, the growth should be removed. In the majority of cases colotomy is unwarrantable.

DR. F. W. MCRAE said that the only cases of stricture of the rectum he had seen were due to syphilis. He reported a case where he had performed inguinal colotomy, and had also removed the cancerous growth. This case had been treated for piles. In another case a spasmodic stricture of the urethra was relieved by divulging the sphincter.

DR. M. B. HUTCHINS read a paper on "Mixed Infection." (To be published in this JOURNAL.)

DR. DUNBAR ROY read a paper entitled "Paresis and Paralysis of the External Rectus of the Eye, with Report of Two Cases." In one case the trouble was due to malaria, shown by its yielding to anti-malarial treatment. In the second case the paralysis followed a blow on the temple. A cure was effected by gymnastics and electricity. He reviewed the literature of malarial eye diseases and the paralysis caused by trauma.

DR. FRANK TRESTER SMITH said that he had never known a case of paralysis of the eye muscles to be ascribed to malaria, but the result showed that the conclusion probably was correct.

DR. G. W. DRAKE asked if, in the second case, the paralysis was caused by a hemorrhage, where the hemorrhage was located, in the muscle or in the brain.

DR. ROY thought that there was some effusion in the muscle, and that the center in the brain may have needed some impulse.

DR. G. W. DRAKE read a paper entitled "The Code of Medical Ethics." The basic principles upon which the grand old edifice of medicine are builded are "beneficial and professional liberality," community of knowledge, open medicine, and no therapeutic secrets. He who violates these principles by dispensing secret medicines is a medical heretic or anarchist. He divided all doctors into two classes, regulars and irregulars. He is an irregular who uses any sectarian name or term denoting that his practice is based on any exclusive dogma. He is a regular, whether he prescribes little or large doses, or no doses; whether he employs the principle of "similia" or "contraria," etc., provided he does not assume any ostentatious epithet by which to attract the unwary public, such as eclectic, homeopathic, hydropathic, Indian, hoodoo, etc.

SECOND DAY, WEDNESDAY, OCTOBER 10, 1894.

DR. GEORGE R. WEST read a paper on "Uterine Cancer." (To be published in this JOURNAL.)

DR. W. E. B. DAVIS said that as a rule these cases came to the surgeon too late to remove all the disease. The time for the operation is in the early stage. We cannot be sure that the trouble is confined to the cervix, so that total removal is generally indicated. The microscope, in proper hands, is a valuable aid to diagnosis.

DR. J. M. MATTHEWS said that hysterectomy was not in his line, but cancer was in the line of every surgeon. Cancer is a disease which requires great care in diagnosis. There were cases operated on which should be let alone. He advised that to quiet pain opium should be used, where pain is a factor in the case. He had five cases in which the diagnosis of carcinoma was made by competent microscopists, and this was proven to be a mistake by the subsequent history. He would rather rely on the clinical history.

DR. JOHN A. WYETH said that the hope of the profession lay in early diagnosis. This is the secret of success. Operation is simple at that time, and successful. Cancer is a local disease, the result of irritation. When in doubt it is best to operate.

DR. W. G. BOGART believed the early diagnosis of cancer to be of great importance. In fact, upon the early diagnosis depends our good results. He insisted upon a more thorough study of our cases. Morphia was to be used in these cases sufficiently to overcome pain. We should not neglect to have a microscopical examination made to confirm the general and physical signs we have already had to make us suspect cancer.

DR. W. E. B. DAVIS read a paper on "The Treatment of Stone in the Kidney," in which he pointed out the methods of diagnosis, and dealt with the technique of operating for the relief of the condition produced by stone. He usually makes incision in lumbar region, but if tumor is too large to be removed through incision, extends it toward the median line. He was working on an instrument by which the urine could be collected from each ureter separately without catheterization of the ureters. He reported a case resembling stone in the kidney which was due to ectopic gestation. A nephrotomy should be performed rather than a nephrectomy, if there is much healthy kidney tissue.

DR. J. MCFADDEN GASTON said that while he had met with a number of cases in which urinary calculi had been arrested in the ureter, he had had but little experience in treating stone in the kidney. As Dr. Davis had referred to multiple abscess resulting from stone in the kidney, he would present some points of a case which he had reported before the American Surgical Association in May last, a reprint of which appeared in its transactions. There was trouble connected with the left kidney in a man aged sixty; there was but little sensitiveness upon pressure over the kidney nor were there any signs of pus, but fluctuation extending from the lumbar region in two distinct areas of several inches in breadth, with a space between, not affected, led to the inference of perinephritic abscess. Upon using a trocar in the left hypochondriac region, pus was discharged, and, an incision being made, a drainage tube was left in the opening. Subsequently the area of fluctuation on the line with the umbilicus was treated in the same manner, and pus continued to be discharged from both openings. Realizing that the trouble came from the kidney, after consultation with the doctors in charge, Seltze and Stewart of Marietta, it was determined to cut down upon the left kidney. As a guide in the operation I passed a trocar into the lower part of the pelvis and found pus. Another trocar was introduced three-fourths of an inch above the first, finding pus also. Using this indication, a free incision was made, connecting the two trocars, and a large quantity of pus was discharged, coming in part from the pelvis and in a larger quantity from the perinephritic abscess outside of the kidney. Upon introducing my finger into the pelvis of the kidney, no calculus was discovered. But on washing out the kidney with warm water two small stones were discharged from the entrance of the ureter. The two calculi had each a flat and hemispherical surface, showing that they had been held in contact with each other in the ureter, so as to completely occlude it. This explains the absence of pus from the urine, and it is evident that no urine could have passed through into the bladder, and that inflammation of the kidney completely arrested its action as a gland, causing the suppression of urine in this organ. The perinephritic abscess resulted from the inflammation of the surrounding structures and developed the facial abscess, which involved the abdomen as far as the umbilicus. Drainage was maintained by gauze and a rubber drainage tube for the external opening into the pelvis of the kidney, and there was no further discharge from the abdominal opening. Pus made its appearance in the urine, but the function of the kidney operated upon was very much impaired, and there was but slight evidence of urine being discharged from the external wound. The exhaustion of the patient was such that he died in the course of a month.

DR. A. R. ROBINSON read a paper, "The Importance of Early Treatment in Cutaneous Cancer," illustrating the subject with diagrams. The earlier the treatment, the less tissue to be removed and the greater the chance of cure. The superficial discoid form may disappear, may grow very slowly, or may grow rapidly, and all should be operated on. The papillary may begin as wart or mole, or may be secondary to the superficial discoid. Early treatment is more strongly called for. The deep-seated or nodular should be diagnosed early and removed. It first appears as a small, hard nodule under the skin gradually increasing in size. The deep tissue should be removed.

DR. JOHN A. WYETH said that he did not rely on the knife alone, but used caustics. In the nodular form he would not begin with the paste. He related a case in which recurrence took place after excision three times, and recovery took place after the use of Marsden's paste. In another case, after an attack of erysipelas, he believed the disease due to a germ, and that a cure would be found.

DR. J. MCF. GASTON remarked that he had recently operated at the Southern Medical College for an epithelioma as large as a child's head upon the left side of the face and neck of a man about fifty years old. On conference with Drs. Nicolson and Elkin, it was determined that the danger of hemorrhage rendered it unsafe to use the knife and that the thermo-cautery should be employed. A line of demarcation was made by an incision through the skin, and then the separation of the tumor was effected by the thermo-cautery with comparatively little loss of blood. Though the facial and some smaller vessels spurted when first divided, the repeated touches of the cautery at a dull, red heat arrested all hemorrhage. Dr. Elkin had one instrument, while Dr. Gaston used the other, thus expediting the detachment of the large ulcerated mass from the deeper tissue of the neck. The loss of blood did not exceed one pint. The extensive surface was thoroughly curetted, and again cauterized, but it is doubtful whether all of the diseased structure was removed. Cotton was packed into the wound and covered with iodoform gauze secured firmly by a roller bandage. The dressing had all been changed, and the eschar separated at the end of the first week.

DR. JOHN A. WYETH read a paper on "A Report of Some Rare Surgical Lesions Connected with the Liver," relating two cases of obstruction of the hepatic, cystic, and common ducts with operations; also a case of traumatic rupture of the gall-bladder, with escape of bile in the peritoneal cavity; operation twenty-four hours, later followed by recovery. The torn edges in the gall-bladder were stitched to the edge of the abdominal incision. The fistulous opening closed spontaneously in two weeks.

In another case, which seemed to be carcinoma of the liver, the neoplasm was attached to the margins of an abdominal incision, and allowed to become infected with the ordinary coccus of suppuration. The wound was kept open two months, and the patient improved. The growth did not enlarge. Patient can now attend to business.

He believed that the toxine of erysipelas, as well as that of ordinary suppuration, has a curative effect on epithelioma and sarcoma. In regard to carcinoma, our knowledge is not as yet so positive.

DR. W. E. B. DAVIS said that cases of obstruction of biliary ducts should be operated on rapidly—can't stand long operation. The best method is incision, the insertion of a tube, and packing with gauze. Do not take time to sew up duct.

DR. J. MCF. GASTON indorsed external drainage, as advocated by Dr. Davis. In a patient which he had seen in consultation at Birmingham, Ala., who was in a state of collapse, with great distention of the abdomen, and sensitiveness, indicating general peritonitis, an incision was made below the points of the rib in the region of the gall-bladder. So soon as the peritoneal cavity was opened a dirty, bile-stained fluid escaped; the gall-bladder was found to be ruptured. The cavity was thoroughly washed out with hot sterilized water and strips of iodoform gauze packed from the gall-bladder to the surface for drainage. The patient died three hours afterward.

DR. T. ELLIS DREWRY read a paper on "Burns, and Treatment Thereof," in which he advocated antiseptics. The indications are to allay irritation, reduce pain, prevent sepsis.

DR. W. G. BOGART said that no class of injuries are more seriou or difficult to obtain good results from than bad burns.

The steps indicated in the treatment of burns are :

1. Relief of pain.
2. Overcome shock.
3. Dressing injury.

First indication is met with hypodermic injection of morphine.

Second, with strychnia or alcoholic stimulants.

Third, with white lead, iodoform with vaseline, etc.

DR. J. B. S. HOLMES delivered the President's address—"Some Causes Leading to Invalidism in Women."

He protested against the amount of work required of girls at school. The fault lies with the parents, who want them to graduate at an early age. The system sends cases to the doctor. He suggested that the girl be treated as a boy until the time of menstruation. The mother should inform the daughter of the expected change. She should be taught to keep quiet during the flow three or four years.

Among the causes he mentioned, the study of music, high heels, exposure at time of menstruation, infection of childbirth from failure to repair a tear, or from applications by the surgeon, gonorrhea.

On motion, the society voted that its thanks be extended to the retiring President for his excellent address.

DR. ARTHUR G. HOBBS read a paper entitled "Adenoids and Their Treatment with a New Forceps." The means used in treatment are the ring knife of Meyer, the finger, which is dangerous on account of the liability of getting material in larynx, Volkman's curette, the gouge, wire loop, and forceps, which is the most rational method. He represented an original forceps for the removal of these growths.

DR. J. A. GOGGANS presented a specimen of extra-uterine pregnancy. The case, when first seen, was diagnosed as extra-uterine pregnancy with ruptured sac, but operation was refused until a month later. Abdomen was full of blood. Sac firmly adherent in pelvis, hemorrhage controlled by gauze packing. Death took place thirty-six hours later.

At night the Society attended a reception given by the medical profession of Atlanta, at the Capital City Club.

THIRD DAY, THURSDAY, OCTOBER 11, 1894.

The Society witnessed an operation by DR. JOHN A. WYETH, of New York, for the removal of a large cyst of the thigh, at the Southern Medical College, after which it was called to order at the Kimball House.

DR. VALENTINE H. TALIAFERRO read a paper on "Transfusion of Blood and Infusion of Salines." He advised the operation for hemorrhage and also for shock. He used a saline solution heated to 110° F. He had operated five times and had seen a number of cases, and had never seen any harm. He reported cases where the measure was successfully used, where death seemed imminent.

DR. WM. PERRIN NICOLSON read a paper entitled "Combination of Carbolic Acid and Camphor as an Antiseptic and Local Anesthetic." He recommended it for all wounds, for pruritus and for parasitic diseases. It causes no reaction and there is no absorption. Catgut was kept soft and aseptic a year in the mixture.

DR. R. R. KIME said that he had used the following preparations of camphor and carbolic acid. Camphorated phenol is made of pure carbolic acid one part, to one or two parts gum camphor. Has used camphorated phenol for years, considers it a positive germ destroyer. It checks putrefaction at once, as well as pus formation. It also checks septic invasion when it can be brought in contact with the germs and foci of infection. It is the best remedy we have to apply to uterine cavity after curetting or removal of putrid-septic or purulent material, swabbing out with cotton, and on gauze packing. It instantly checks the putrefaction, etc. It is the best remedy to check pus formation in all suppurating wounds, and is excellent in burns, erysipelas, carbuncle, and dressing wounds. Prefers the name camphorated phenol in contradistinction to the proprietary preparation known by the name camphophenique. Had published an article about one year ago setting forth the combinations and uses of gum camphor and carbolic acid.

Dr. R. P. Johnson read a paper on "Headaches; Their Etiology and Treatment." He maintained that headaches are especially important in childhood. As causes, he mentioned over-loaded stomach, constipation, exposure in the hot sun, sedentary habits, uterine and ovarian diseases. Treatment, if stomach is full, free emesis; if bowels are torpid, large enemas followed by saline cathartics; if head is hot, apply cold; if feet are cold, apply heat. Careful diet is always indicated.

The Society adjourned to meet next October in Chattanooga. Following are the officers elect:

President, R. M. Cunningham, Birmingham, Ala.; First Vice-President, J. C. LeGrand, of Alabama; Second Vice-President, Floyd W. McRae, of Georgia; Third Vice-President, G. W. Drake, of Tennessee; Secretary, Frank Trester Smith, Chattanooga; Treasurer, George R. West, Chattanooga.

---

### The Indications for Bleeding.

Blood-letting is now a lost art. Probably very few of the younger generation of doctors ever saw the little operation done. Sir Benjamin Richardson is still an advocate of it, and gives the following indications (*Aesclepiad*):

In acute spasmodic seizures, as in spasms of croup, in colic, and in angina with symptoms of oppression of the right side of the heart with blood.

In acute pain, membranous or spasmodic, as in sudden pleuritic or peritoneal pain, or in pain from passage of a calculus, hepatic or renal.

In acute congestions of vascular organs, as of the lungs or brain, apoplexies.

In cases of sudden shock or strain, as after a fall or blow, sunstroke, or a lightning shock.

In some exceptional cases of hemorrhage of an acute kind, unattended by pyrexia.

